# *dRecQ4* Is Required for DNA Synthesis and Essential for Cell Proliferation in *Drosophila*

**DOI:** 10.1371/journal.pone.0006107

**Published:** 2009-07-02

**Authors:** Yanjuan Xu, Zhiyong Lei, Hai Huang, Wen Dui, Xuehong Liang, Jun Ma, Renjie Jiao

**Affiliations:** 1 State Key Laboratory of Brain and Cognitive Science, Institute of Biophysics, the Chinese Academy of Sciences, Beijing, China; 2 Graduate School of the Chinese Academy of Sciences, Beijing, China; 3 Divisions of Biomedical Informatics and Developmental Biology, Cincinnati Children's Hospital Research Foundation, Cincinnati, Ohio, United States of America; University of Minnesota, United States of America

## Abstract

**Background:**

The family of RecQ DNA helicases plays an important role in the maintenance of genomic integrity. Mutations in three of the five known RecQ family members in humans, *BLM*, *WRN* and *RecQ4*, lead to disorders that are characterized by predisposition to cancer and premature aging.

**Methodology/Principal Findings:**

To address the *in vivo* functions of *Drosophila RecQ4* (*dRecQ4*), we generated mutant alleles of *dRecQ4* using the targeted gene knock-out technique. Our data show that *dRecQ4* mutants are homozygous lethal with defects in DNA replication, cell cycle progression and cell proliferation. Two sets of experiments suggest that *dRecQ4* also plays a role in DNA double strand break repair. First, mutant animals exhibit sensitivity to gamma irradiation. Second, the efficiency of DsRed reconstitution via single strand annealing repair is significantly reduced in the *dRecQ4* mutant animals. Rescue experiments further show that both the N-terminal domain and the helicase domain are essential to dRecQ4 function *in vivo*. The N-terminal domain is sufficient for the DNA repair function of dRecQ4.

**Conclusions/Significance:**

Together, our results show that *dRecQ4* is an essential gene that plays an important role in not only DNA replication but also DNA repair and cell cycle progression *in vivo*.

## Introduction

*RecQ4* encodes a DNA helicase that belongs to the RecQ family; in humans, this family consists of five members [1]–[5]. Unlike other RecQ family members such as BLM and WRN [6]–[12], the biological functions of RecQ4 remain relatively less clear and more controversial [13]–[25]. For example, various studies have led to contradictory conclusions on where RecQ4 is localized [10], [25], [26]. Furthermore, the sensitivity of *RecQ4* deficient cells or organisms to treatments that block DNA replication or cause DNA damage, e.g., ionizing radiation, remains poorly resolved [27]–[29].

Cancer predisposition of either human patients or mice models with *RecQ4* mutations represent another unresolved issue (for review, see [2]). Mutations in the human *RecQ4* gene have been found to contribute to three rare syndromes: Rothmund-Thomson syndrome [5], [30]–[32], RAPADILINO syndrome [22], [25] and Baller-Gerold syndrome [2], [23]. Currently there is no common conclusion on whether these three syndromes are independent disorders or represent one syndrome with different symptoms. Several labs have developed mice models with different RecQ4 mutations, but these mice show different phenotypes that range from embryonic lethality to defects restricted to adult mice, some of which resemble the symptoms of human patients [19], [28], [33].

Several recent studies have revealed new insights concerning the role of RecQ4 in DNA replication initiation [18], [20], [21], [24]. Cut5, the metazoan homolog of *Saccharomyces cerevisiae* Dpb11, which is required for loading DNA polymerases onto chromatin, was shown to interact with the *Xenopus* RecQ4 (xRecQ4) both *in vitro* and *in vivo* [20], [21]. Purified N-terminal fragments of xRTS/xRecQ4 were able to rescue the DNA replication defects of xRecQ4-depleted *Xenopus* egg extracts [20]. In mammalian cells, RecQ4 has been shown to interact with RAD51 and PARP1, suggesting that it may also participate in DNA repair [2], [10], [34], [35]. However, the role of RecQ4 in DNA repair has not been fully characterized, particularly in the context of an *in vivo* system.

Unlike in mammals, the fruit fly genome encodes three complete RecQ helicases, namely dBLM, dRecQ4 and dRecQ5 [24], [36]–[43]. In addition, DmWRNexo was recently identified as the *Drosophila* homologue of human WRN exonuclease domain [44], [45]. In order to develop a model system more amenable to genetic analysis of RecQ4 function *in vivo* which would also help to clarify, at least, some of the controversies about RecQ4, we set out to characterize RecQ4 in *Drosophila*.

In this report, we describe the generation and phenotypic analyses of *dRecQ4* mutants in *Drosophila*. Our results show that the *dRecQ4* mutants exhibit defects in DNA replication. They are also selectively sensitive to paraquat and gamma irradiation. Mutant animals exhibit lower efficiency of double strand break (DSB) repair as assayed by reconstitution of the DsRed transgene *in vivo* [46]. Rescue experiments with various truncated dRecQ4 proteins suggest that the N-terminal domain of dRecQ4 is essential for DSB repair, whereas both the N-terminal domain and the helicase domain are indispensable for DNA replication and animal viability.

## Results

### *dRecQ4* is essential for development

**Figure 1:**
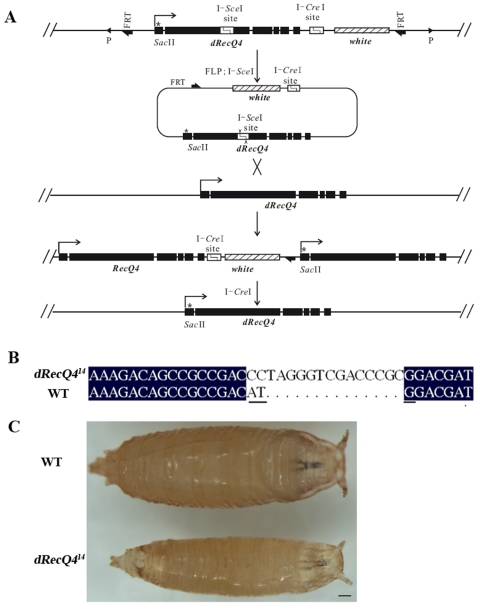
Generation of *dRecQ4^14^* mutants: strategy and identification. (A) Schematic view of the *dRecQ4* locus and targeting strategy. A transgene containing a mutant *dRecQ4* and the marker gene *w+* is circularized from the genome by FLP recombinase and linearized by the yeast restriction endonuclease I-*Sce*I. Alignment of the targeting DNA and the resident *dRecQ4* locus by ‘ends-in’ recombination results in a duplication of *dRecQ4*. Then the genomic DNA is cut by another rare-cutter I-*Cre*I, repaired by homologous recombination, leading to a single copy of *dRecQ4*. * indicates mutation of the start codon. (B) Sequence comparison of the mutant (*dRecQ4^14^*) and the wild type (WT) indicates that *dRecQ4^14^* harbors the expected changes as was designed. The translation start codon ATG (underlined in wild type sequence) is disrupted and the open reading frame is also shifted for *dRecQ4^14^* mutant. (C) *dRecQ4^14^* mutants are homozygous lethal and die at early pupal stage when raised at 25°C. A *y w* pupa, serving as a wild type control, and a *dRecQ4^14^* mutant are shown. Scale bar = 150 µm

Prior to the report of Wu et al., there were no transposable elements inserted within or nearby the *dRecQ4* locus [24]. We took advantage of the targeted knockout technique to generate *dRecQ4* mutants through the replacement of the endogenous locus with an engineered mutant form via homologous recombination. Specifically, an 8 kb genomic fragment was modified by replacing the start codon ATG with CCTAGGGTCGACCCGCG and inserting an I-*Sce*I recognition site into the second exon of *dRecQ4* (Figure 1A; see Materials and methods for details). Targeting of the *dRecQ4* locus was achieved by a modified procedure described by Rong and Golic [47] and Egli and colleagues [48], [49]. Four mutant candidates were obtained and confirmed by restriction enzyme digestions; all four alleles showed similar phenotypes in the viability test (see below). One of these alleles, *dRecQ4^14^*, was further confirmed by DNA sequencing (Figure 1B) revealing that the start codon mutation and open reading frame shift are as designed. This allele, which can be fully rescued by a genomic rescue transgene as judged by adult flies' viability (see below), was used for detailed phenotypic analysis throughout this study.

*dRecQ4^14^* mutants are homozygous lethal, indicating that *dRecQ4* is an essential gene. Using a GFP marked balancer chromosome, we separated the homozygous from the heterozygous *dRecQ4^14^* animals. Nearly all the homozygous mutants survive for up to 8 days under normal culture conditions. However, they exhibit developmental delays when compared with heterozygous siblings or *y w* wild type flies and eventually die at early pupal stage (Figure 1C). The lethal phenotype of the *dRecQ4^14^* mutants can be fully rescued by either a genomic fragment of *dRecQ4* or a UAS mediated *dRecQ4* expression (data not shown), further indicating that the lethality phenotype was a direct consequence of the *dRecQ4* mutation.

To determine the role of maternal contributions to development, we set out to generate *dRecQ4* germline clones in females that carry *FRT* combined *dRecQ4* mutation and *ovo^D^* chromosomes (Table 1). However, when these females were crossed to *y w*; *dRecQ4^14^/TM3, Kr-GFP* males, no eggs were obtained (Table 1). The oocytes from *dRecQ4^14^ FRT2A/FRT2A ovo^D^* females failed to go beyond stage 6 of oogenesis (data not shown). These results suggest that *dRecQ4* is essential for oogenesis, further supporting the conclusion that *dRecQ4* is an essential gene critical to cell viability.

**Table 1:** Statistics of germline clone analysis.

Maternal genotypes	Number of mothers tested	Number of mothers with eggs
*FRT2A/FRT2A ovo^D^*	59	12
*dRecQ4^14^ FRT2A/FRT2A ovo^D^*	85	0

### *dRecQ4* loss-of-function affects endogenous DNA replication both in the salivary glands and the late larval brain

**Figure 2:**
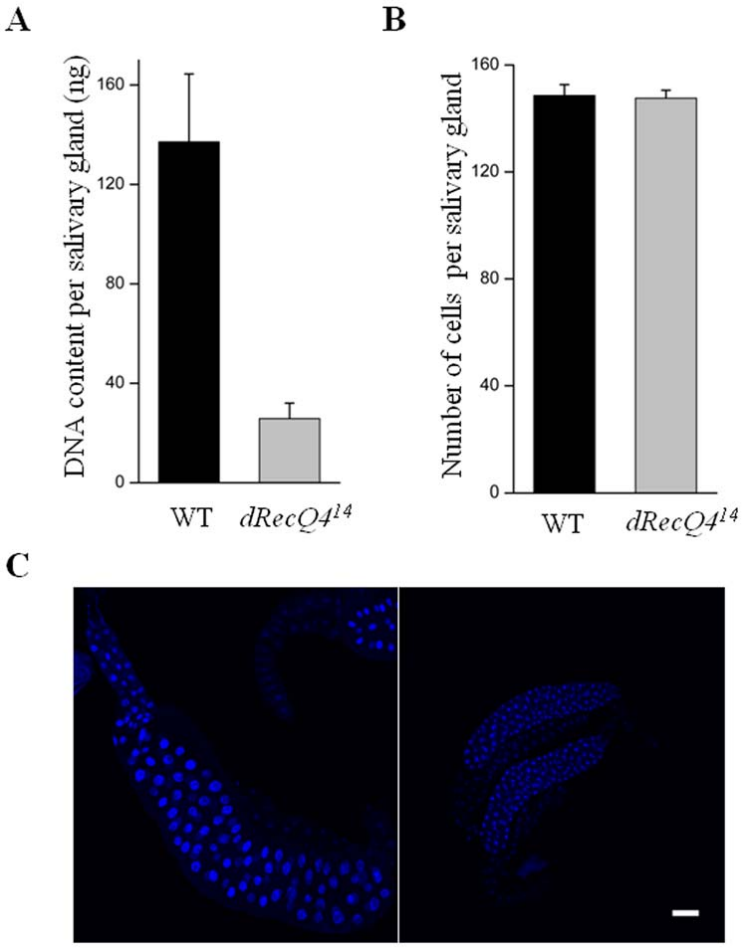
*dRecQ4* mutant cells contain less genomic DNA than wild type cells. (A) DNA content of the salivary glands of *dRecQ4^14^* mutants is lower than that of wild type. P<0.05. (B) The cell number of salivary glands from *dRecQ4^14^* mutants and wild type remains unchanged. P<0.001. Error bars represent the standard deviation of the mean value of three independent experiments. (C) 5 days old salivary glands from wild type and the mutant larvae were stained with DAPI. Note that the mutant nuclei are smaller. Scale bar = 100 µm

It has been shown in *Xenopus* egg extracts that DNA replication is blocked when RecQ4 is depleted [20], [21]. To specifically determine whether *dRecQ4* is essential for DNA replication *in vivo*, we measured both DNA content and cell numbers of salivary glands from wild type and *dRecQ4* mutant animals. Figure 2B shows that salivary glands from wild type and mutants have similar cell numbers at third instar larval stage (5 days AED). However, the total amount of DNA from each salivary gland at this stage differs significantly between wild type and mutants (Figure 2A). The amount of DNA normalized by cell number is much lower in mutant cells than in wild type cells (∼0.18 ng/cell and ∼0.92 ng/cell, respectively), indicating a defect in DNA accumulation, presumably reflecting an under replication of DNA. Salivary gland increases its DNA content through cell cycle independent endoreplication. Inefficient DNA endoreplication of salivary gland cells is also consistent with the finding of small cells and nuclei in this tissue of mutant animals (Figure 2C).

**Figure 3:**
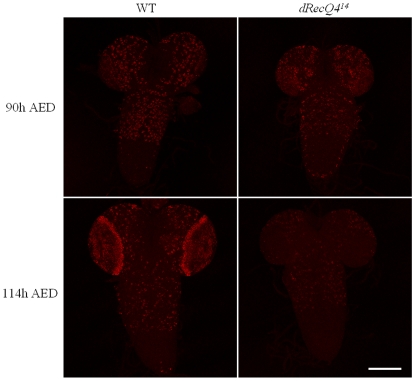
Incorporation of BrdU is significantly reduced in *dRecQ4* mutants as compared to wild type. The incorporation of BrdU was visualized by staining with anti-BrdU. As shown in the upper panel, at 90 hours AED, the BrdU incorporation in the brain is only slightly different while at 114 hours AED, mutant larvae incorporate significantly less BrdU as shown in the lower panel. Scale bar = 100 µm

To directly investigate whether *dRecQ4* is required for DNA replication, particularly in the non-endoreplicating cells, we performed BrdU labeling followed by anti-BrdU immuno-staining on the larval brain. Figure 3 shows that at the stage of four days after egg deposition (AED), wild type and *dRecQ4* mutants have comparable BrdU incorporation likely reflecting maternal contributions of dRecQ4 protein in the mutants. However, at five days AED the mutant brain incorporates much less BrdU than the wild type, which is consistent with the observed reduction of DNA accumulation in salivary glands (see above). Our data are consistent with those of Wu (Wu et al., 2008) and together they demonstrate that *dRecQ4* is involved in DNA replication in *Drosophila*.

### *dRecQ4* is involved in double strand breaks repair

**Figure 4:**
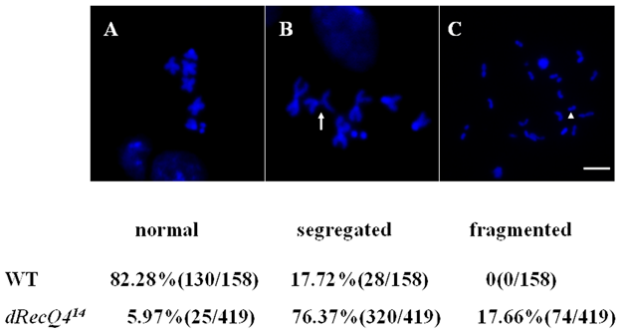
Chromosomal aberrations in *dRecQ4^14^* mutant cells. Mitotic chromosome patterns of wild type and *dRecQ4^14^*mutant larval brains are shown in (A), (B) and (C). (A) Normal pattern of metaphase chromosomes for wild type cells. (B) and (C) show typical metaphase chromosomes of *dRecQ4^14^* mutant cells. In (B), sister chromatids are precociously separated, while in (C) chromosomes are mostly broken into smaller fragments. The percentage of cells for the three categories in wild type and *dRecQ4^14^* mutants is shown below. Total cell numbers analyzed in each case are indicated in parenthesis. Scale bar = 5 µm

Inactivation of *RecQ4* in mouse results in defective sister chromatid cohesion and aneuploidy [50]. To determine whether *dRecQ4* deficiency causes a similar effect on chromosomal behavior in *Drosophila* cells, we analyzed metaphase spreads from wild type and mutant brain cells. In *dRecQ4* mutants, the spreads' patterns fall into three major categories (Figure 4A–C): normal pattern (Figure 4A), segregated (Figure 4B) and fragmented (Figure 4C) aberrant patterns. The segregated patterns are indicative of a failure of sister chromatids association (arrows in Figure 4B). The fragmented patterns have broken chromosomes, often with the broken ends fused together (arrow heads in Figure 4C). Statistical analysis shows that the frequency of aberrant patterns in *dRecQ4^14^* mutants is much higher than in wild type control. Specifically, mutant cells have less than 10% of normal patterns, with the aberrant segregated and fragmented patterns representing the majority of the mitotic cell population. In wild type cells, over 80% of the cells have normal patterns, with only less than 20% being the mildly abnormal segregated patterns. These results suggest that *dRecQ4* is important for maintaining genome integrity.

To investigate DNA repair pathways in which *dRecQ4* participates, we treated mutants with various DNA-damaging mutagens including hydroxyurea (HU), methyl methane sulfonate (MMS), paraquat and gamma irradiation (Table 2). These mutagens exert their effects through distinct mechanisms and, thus, can provide insights into DNA repair defects in *dRecQ4* mutants. Our results show sensitivity of *dRecQ4^14^* mutants to paraquat and gamma irradiation (Table 2). Paraquat mainly causes single-base damage which is corrected through base excision repair pathway. Using T7 phage display screen, human RecQ4 was found to interact with poly(ADP-ribose) polymerase-1 (PARP-1), an enzyme that maintains genome stability through its involvement in base excision repair pathway ([34]). Together with our in vivo data, they demonstrate that RecQ4 is involved in the base excision repair pathway. DNA double strand break (DSB) is the major type of DNA damage after gamma irradiation, the sensitivity of *dRecQ4^14^* mutants to gamma irradiation suggests that *dRecQ4* is also involved in the DSB repair pathways.

**Table 2:** Sensitivity of *dRecQ4^14^* mutant flies to mutagens.

Mutagen	N. hetero./N. homo.	Relative survival	*dRecQ4^14^* mutant response
Nothing	2.12(403/190)	94.3%	N.A.
HU (6.4 mM)	2.11(327/155)	94.8%	not sensitive
MMS (0.1%)	2.24(470/209)	88.9%	not sensitive
Paraquat (10 mM)	3.77(490/130)	53.1%	sensitive
Gamma irradiation (9 Gy)	2.91(918/315)	68.6%	sensitive

See Materials and methods for details of the experiment.

**Figure 5:**
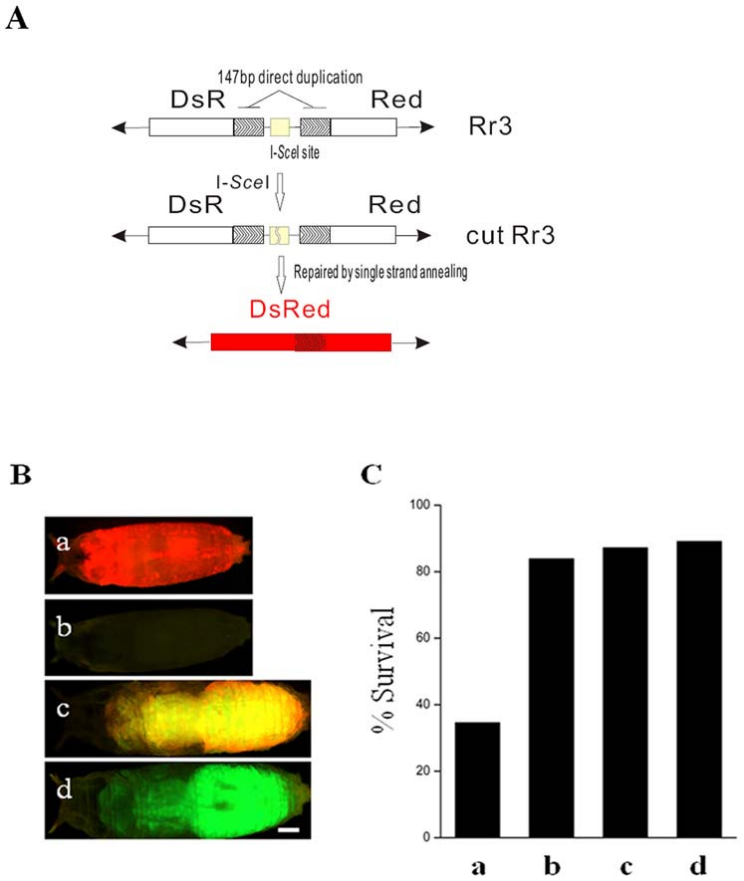
Repair efficiency of DSBs in *dRecQ4^14^* mutants is lowered. (A) The repair reporter construct, Rr3, consists of a *DsRed* gene with an I-*Sce*I recognition site in the middle. Flanking the cut site is a 147 bp direct duplication of a part of the *DsRed* gene sequence. The modified *DsRed* gene is put into a P element. The entire Rr3 does not express a functional DsRed protein, however, when a DSB is generated by the I-*Sce*I enzyme, repair through the single strand annealing (SSA) pathway results in a functional *DsRed* gene. A GFP marked balancer chromosome was used to separate the homozygous (panel B, a and b) from the heterozygous (panel B c and d) *dRecQ4^14^* animals. The heterozygous mutants served as control. (C) When Rr3 is not cut, control (d, GFP^+^ and DsRed^−^) and homozygous *dRecQ4^14^* mutant (b, GFP^−^ and DsRed^−^) animals exhibit similar survival ratios; when Rr3 is cut, the survival ratio is significantly reduced in *dRecQ4* mutants (a, GFP^−^ and DsRed^+^) compared with the control (c, GFP^+^ and DsRed^+^). The relative survival ratio (number of pupae/number of first instar larvae) of each category is shown in (C). More than 120 animals were counted for each category. Scale bar = 250 µm

To demonstrate more directly that *dRecQ4* participates in DSB repair, we employed the *in vivo* inducible DSB break-repair system in *Drosophila* (Fig. 5A; [46]). In this system, the reporter construct, *Rr3*, consists of a DsRed gene interrupted by the recognition sequence for the rare-cutting endonuclease, I-*Sce*I. UIE is a transgene that expresses the enzyme I-*Sce*I under the control of the ubiquitin gene promoter. The intact *Rr3* element does not express a functional DsRed gene product owing to the presence of the cutting site. However, when a DSB is formed at the I-*Sce*I cutting site, repair via the single-strand annealing pathway results in a functional DsRed gene (Figure 5B a and c). We tested the repair efficiency of the induced DSBs both in the presence and in the absence of *dRecQ4^14^* mutation. When *Rr3* is not cut, the heterozygous control and homozygous mutant animals have similar survival ratios (Figure 5C. category b and d), which serves as a system control. However, when *Rr3* is cut, the survival ratio is significantly reduced in *dRecQ4* homozygous mutant background compared with heterozygous animals (Figure 5C. category a and c), indicating a reduced efficiency of DSB repair in the absence of *dRecQ4* gene function. These results provide additional support to our conclusion that *dRecQ4* is involved in DSB repair *in vivo*.

### *dRecQ4* loss-of-function leads to mitotic (M) phase arrest and reduction of cell proliferation in late wing and eye imaginal discs

**Figure 6:**
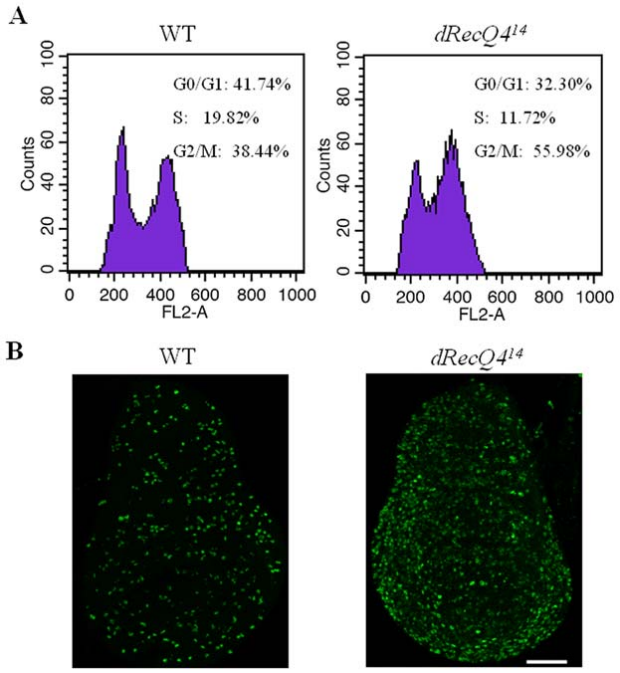
The wing imaginal discs of the *dRecQ4* mutants have more M phase cells than wild type. (A) FACS analysis showing cell cycle profiles of the wing discs of WT and *dRecQ4^14^* animals. *dRecQ4^14^* mutant discs have more G2/M phase cells at the expense of G1 and S phase cells. (B) WT and *dRecQ4^14^* third-instar larvae stained with anti-phospho histone H3 (Ser 10) antibody (mitotic marker). Note that mutant wing discs exhibits higher levels of M phase cells compared with wild-type discs. Scale bar = 100 µm

Cell cycle progression is strictly controlled by a strong checkpoint system that arrests progression of the cell cycle until either DNA replication is completed or DNA damage is repaired [51]. To test whether DNA replication and/or repair defects in *dRecQ4^14^* mutants lead to cell cycle arrest *in vivo*, wing imaginal discs from wild type and mutant larvae were dissected, trypsinized and stained with propidium iodide (PI), followed by fluorescence activated cell sorting (FACS). Compared with wild type, more cells from *dRecQ4^14^* mutants are accumulated at G2/M phase at the expense of G1/G0 and S phase cells (Figure 6A). It is notable that the second peak is shifted with its position closer to the G1 peak in the *dRecQ4* mutant cells comparing with that in the WT cells (Figure 6A). A possible explanation could be that in the absence of dRecQ4, the S-M checkpoint becomes defective, which allows the mutant cells to enter mitosis with incompletely replicated DNA. Immunostaining using antibody against phospho-histone H3, which serves as M phase marker, shows a significant increase of M phase cells in the wing discs of *dRecQ4^14^* larvae compared with that of wild type (Figure 6B).

**Figure 7:**
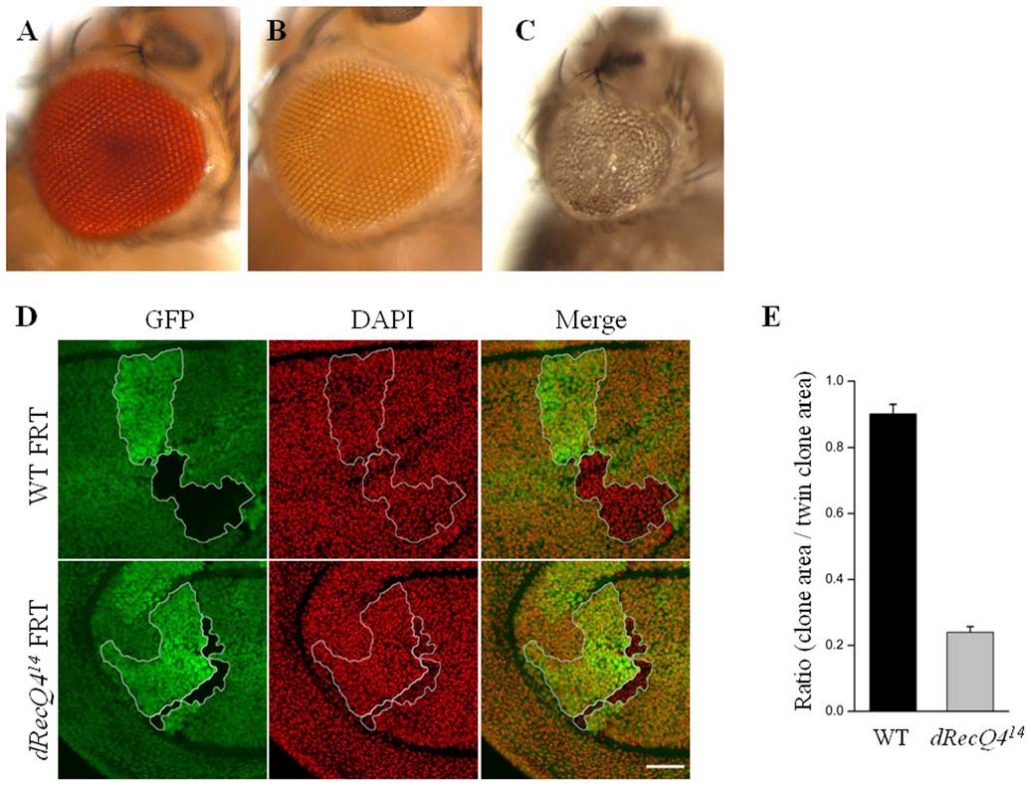
*dRecQ4* mutation affects eye development and cell proliferation in the wing discs. *dRecQ4^14^* mutant flies that are rescued by the *pTARG-dRecQ4[rescue]* transgene have as normal eyes (B) as wild type (A). When crossed to flies that carry *ey-FLP,* the rescuing transgene of *dRecQ4* is removed specifically in the eye, leading to smaller and rough eyes (C). (D) Upper panel, wild type sister clones marked by either absence of GFP (GFP-, dark region) or two copies of the GFP (2XGFP, bright region). Lower panel, sister clones of a homozygous *dRecQ4^14^* mutant clone marked by GFP- (dark region) and a homozygous wild type twin clone marked by 2XGFP. The *dRecQ4^14^* mutant clone has fewer cells than its wild-type twin clone. (E) Statistical analysis indicates that *dRecQ4* mutant clones occupy less area than their wild type twin clones. Error bars represent S.E.M. n = 29. P<0.001. Scale bar = 30 µm

To test whether the cell cycle aberration in the absence of dRecQ4 function leads to cell proliferation defect, two strategies were employed. First, we conducted the tissue specific knock-out of *dRecQ4* function using tissue specific flipase that acts on a FRT-flanking genomic rescue transgene. We generated a transgenic line, *pTARG-dRecQ4*, which harbors a *dRecQ4* genomic segment flanked by two *FRTs* (see Materials and methods for details). This transgene is able to completely rescue the *dRecQ4* mutant animals to adulthood without any observable defects (Figure 7B). Taking advantage of such rescued flies, we used *ey-FLP* to specifically delete the rescuing genomic fragment in the eye-antenna primordia. Figure 7C shows that tissue-specific knockout of *dRecQ4* in the eye leads to small rough eyes with disorganized and fewer ommatidia, phenotypes that are indicative of reduced cell proliferation.

In a second strategy to determine whether cell proliferation is affected in the absence of *dRecQ4*, normal somatic clone analysis was employed (see Materials and methods for details). As shown in Figure 7D, *dRecQ4* mutant clones can be detected adjacent to their twin clones (lower row) 72 hours after the induction of FLP expression by heat shock. Similar to the wild type (GFP^−^) clones (upper row), the total number of *dRecQ4* clones (GFP^−^, lower row) is similar to their twin clones (2XGFP). However, the *dRecQ4* mutant clones (GFP^−^, lower row) are smaller than the 2XGFP twin clones (lower row), while the wild type (GFP^−^, upper row) clones are similar in size to their 2XGFP twin clones (upper row). The *dRecQ4* mutant clones on average occupy about 20% of the territory that their twin clones occupy (2XGFP); in wild type clones, the size of GFP^−^ clones is similar to that of their twin clones (Figure 7E). Taken together, these data suggest that *dRecQ4* mutant cells are aberrant in cell cycle progression, which may have resulted in cell proliferation defects.

### Both the N-terminal domain and the helicase domain of dRecQ4 are essential to its *in vivo* function

**Figure 8:**
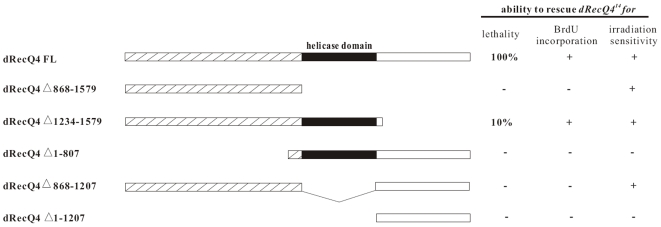
Functional dissection of dRecQ4 protein *in vivo*. Constructs that express full-length and different truncated forms of the dRecQ4 protein are shown. Full length dRecQ4 protein that consists of 1579 amino acids including a helicase domain extending from aa 867 to 1208 can rescue 100% of *dRecQ4^14^* mutants to adulthood. The C terminal deletion form (dRecQ4Δ1234–1579) of dRecQ4 can rescue *dRecQ4^14^* animals to adulthood with an efficiency of only 10%, but fully rescue the BrdU incorporation defects and gamma-irradiation sensitivity of the mutants. None of the other deletion forms can rescue any *dRecQ4* mutant animals to adulthood. However, gamma-irradiation sensitivity can be rescued fully by truncated forms that contain the N-teminal domain, namely dRecQ4Δ868–1579, dRecQ4Δ1234–1579 and dRecQ4Δ868–1207, in addition to the full-length protein.

Our studies described thus far suggest that *dRecQ4* plays a role in multiple processes including DNA replication, DNA repair and cell cycle progression. To clarify which function is primarily responsible for its essentiality and the protein domain for such function, we performed dRecQ4 functional domain dissection experiments. The dRecQ4 protein consists of 1579 amino acids including a helicase domain extending from aa 867 to 1208. A series of deletion mutants of the *dRecQ4* coding sequence were generated, and the resulting truncated proteins are as shown in Figure 8. The corresponding transgenic flies were generated and analyzed for their ability to rescue the mutants' phenotypes including lethality, BrdU incorporation and sensitivity to DNA damaging reagents. The full length dRecQ4 is able to fully rescue *dRecQ4^14^* mutant to adulthood without any obvious defects. The C-terminal deletion form, dRecQ4[Δ1234–1579], can also rescue *dRecQ4^14^* animal to adulthood, but only at an efficiency of about 10%. Nevertheless, it can fully rescue the BrdU incorporation defects and the sensitivity to gamma irradiation of the mutant. Neither of the other truncations, lacking either the N terminal or the helicase domain or both, exhibited any ability to rescue the mutant animals to eclosed flies (Figure 8). However, gamma irradiation sensitivity can be rescued fully by truncated forms that contain the N-teminal domain, namely dRecQ4Δ868–1579, dRecQ4Δ1234–1579 and dRecQ4Δ868–1207, in addition to the full-length protein. These results indicate that both the N-terminal and helicase domain of dRecQ4 are indispensable for animal viability, although the N-terminal domain alone is sufficient to rescue the mutants' sensitivity to gamma irradiation.

## Discussion

The studies described in this report demonstrate that *dRecQ4* is essential for *Drosophila* development. Loss-of-function of *dRecQ4* leads to a failure of proper DNA replication and inappropriate cell cycle progression. The *dRecQ4* mutant animals show a preferential sensitivity to gamma irradiation. Because gamma irradiation mainly causes DNA double strand breaks, this suggests an *in vivo* role of *dRecQ4* in DSB repair, which is further supported by DsRed reconstitution experiment *in vivo*. While the C-terminal domain of dRecQ4 is not essential to its function, the N-terminal domain and the helicase domain are indispensable *in vivo*. Considering our combined results and what is known up-to-date about RecQ4, the following issues are worth further discussion.

(1) *dRecQ4* is the only essential RecQ helicase in flies. The three *Drosophila* helicase genes, *dBLM*, *dRecQ4* and *dRecQ5*, as well as the *Drosophila* homologue of human WRN exonuclease gene, *dWRNexo*, have now all been studied genetically [37], [38], [40], [45] (Chen and Jiao, unpublished). Interestingly, among all the mutants of these *dRecQ* genes, only *dRecQ4* mutants exhibit a homozygous lethal phenotype; mutants of the other *dRecQ* family members only show either fertility defects or various defects in DNA repair, including recombinational repair. There are several possibilities why *dRecQ4* mutants show a more severe phenotype (i.e., lethality) than the other *dRecQ* mutants. First, *dRecQ4* mutants clearly have defects in DNA replication. The defects occur not only in multiploid cells during endoreplication, but also in normal diploid cells. Interestingly, endoreplication defects do not result in total cell number changes although it does affect cell growth, while replication defects in normal diploid cells lead to cell proliferation abnormality. The simplest explanation for this observation is that, at late embryonic stages/early larval stage, the maternal contribution of *dRecQ4* helps the mutant animal acquire proper cell number of the salivary glands before loss of zygotic *dRecQ4* takes effect. However, by the time of second and third instar larval stages when the cells in the wing imaginal discs proliferate extensively, the maternal dRecQ4 has been diluted and/or degraded to a level that is insufficient to promote cell cycle progression (Figure 6A). The failure of proper cell cycle progression and proliferation is likely to be the cause of lethality.

If the DNA replication defects were the primary cause of animal lethality, one would expect that a dRecQ4 domain that is responsible for DNA replication should rescue the lethality. However, our results show that the N-terminal domain of dRecQ4, dRecQ4△868–1579 (Figure 8), which is homologous to the xRecQ4 N-terminal domain, necessary and sufficient for DNA replication in *Xenopus* [20], [21], does not rescue the animal's lethality. As shown in this study, *dRecQ4* also plays an important role in DSB repair (Figure 5). If the DNA repair defects were the primary cause for lethality of *dRecQ4* mutants, one might expect that the other fly *RecQ* mutants that have DSB repair defects, such as *dBLM*, would also be lethal, which in fact is not the case [38]. Together, these considerations suggest that the lethality of the *dRecQ4* mutants is likely a consequence of loss of both of its primary functions, namely DNA replication and DNA repair.

(2) *RecQ4* deficient cells and animals show differential sensitivity to genotoxic agents. It has been controversial in the literature regarding the mutagen sensitivity for *RecQ4* deficient cells and/or model organisms [4], [15], [26]–[28], [34], [52]–[54]. For example, it has been shown by two independent groups that *RecQ4* is involved in UV-induced damage repair in human cells [15], [52]. However, using fibroblasts derived from different RTS patients, Cabral et al. found that these cells are not sensitive to a wide variety of genotoxic agents including ionizing or UV irradiation, H2O2 and HU [53]. Jin et al. showed very recently that *RecQ4* deficient human cells have increased sensitivity to HU, camptothecin (CPT) and doxorubicin (DOX), modest sensitivity to UV or ionizing irradiation[27]. Werner et al. showed that RecQ4-deficient human cells are hypersensitive to oxidative stress such as H2O2 [26], while Woo et al. found changes in the subcellular localization of RecQ4 after exposure to oxidative stress and identify an interaction of RecQ4 with PARP-1 [34]. At the animal level, the mouse model generated by Hoki et al. which bears in frame deletion of exon 13 *RecQ4* shows normal sensitivity to IR and UV irradiation [28]. However, our *Drosophila RecQ4* mutants are strongly sensitive to ionizing radiation, which suggests a role for *dRecQ4* in DSB repair that is in agreement with what has been found in *Xenopus* [54]. The possible explanations for the conflicting sensitivity results of *RecQ4* mutants reported thus far could be as follows: (i) it is possible that *RecQ4* plays a more important role in DSB repair pathways in flies than in humans. There are five RecQ helicases in humans while there are only three in flies. The functions for RecQ members may be more specialized in humans than in flies. For example, in humans BLM and WRN are primarily involved in different DSB repair pathways with BLM more in homologous recombinational repair and WRN more in non-homologous end joining repair [4], [6]–[8]. Since there is only *dWRNexo* in flies, the homologous function of human WRN in flies may have been incorporated in dRecQ4 protein; (ii) more likely, cells derived from different human patients with different mutations or the same mutations in different genetic backgrounds could be also the causes of differential sensitivities. Xu and Liu recently found that the N terminal region and helicase domain of human RecQ4 both possess helicase activity [55], which argues for the possibility that human patients or mutant mouse with intact N terminal region have less severe sensitivity to mutagens. This is very well evidenced by our domain dissection study in *Drosophila*. The N terminal domain is sufficient to rescue *dRecQ4* mutant's sensitivity to gamma irradiation.

(3) Although we expected *dRecQ4^14^* allele to be null based on the designed mutations of the translation start codon ATG and the frame shift of the coding sequence, the phenotypes are generally weaker than Wu's null mutant; for example, our mutants die at early pupal stage, but theirs at early larval stage. It is possible that our mutant allele is not completely null that may express a truncated form of dRecQ4; we note that the 1017th codon (for methionine) is followed by a 562 amino acids in-frame reading frame of dRecQ4 (there is currently no appropriate antibodies available to detect this possible truncated protein of dRecQ4). Unlike the null allele of Wu that could not generate mutant somatic clones in the wing discs, our mutant is capable to support limited cell proliferation (Figure 7D). Our mutant may thus represent a potentially useful tool in further mechanistic studies of DNA repair *in vivo*.

(4) Functional domain dissection combined with rescue experiments suggests that the essential functions of the dRecQ4 protein reside in the N-terminal and the helicase domain. A very recent report by Xu and Liu [56] has shown for the first time that human RecQ4 exhibits dual DNA helicase activity. Two distinct regions of the protein, the conserved helicase motifs and the Sld2-like N-terminal domain, display independent ATP-dependent DNA unwinding activity. Although the N-terminal domain of RecQ4 is sufficient for DNA replication initiation in *Xenopus* ([20]), our *in vivo* data clearly suggests the helicase domain is required for proper DNA replication in *Drosophila*. The C-terminal domain of dRecQ4 is dispensable for its essentiality, but the rescue efficiency of the truncated protein that lacks the C-terminal is only about 10% compared with the full length dRecQ4 protein. It is possible that the C-terminal domain modulates the protein activity of dRecQ4, possibly via amino acids modifications and/or interactions with other proteins.

## Materials and Methods

### Ethics statement

N/A.

### DNA constructs

5 kb *dRecQ4* genomic fragment (coding region of the gene) and 3 kb 5′ regulatory sequences with intended modifications were cloned in the *pTARG* vector [49] to make the gene targeting construct,*pTARG-dRecQ4*. Changes were introduced by PCR with the following oligos (altered bases for either restriction sites and/or mutations are highlighted by underlining). The primers used to amplify the 5 kb *dRecQ4* genomic sequence were 5′-TCCCCGCGGACGATTCGGTGTTCAAGCTAAAAT-3′ and 5′-GGACTAGTGCAGGATGCGATTGAAATCCACTT-3′. The primers for amplifying the upstream 3 kb fragment were 5′-ATAAGAATGCGGCCGCGCTCTCCATCGTGATGGGCCT-3′ and 5′-GGCC TAGGGTCGGCGGCTGTCTTTAATTGTCAATA -3′. Mutation of ATGG to CCTAGGGTCGACCCGCGG generates a new restriction site (*Sac*II) for identification of mutant DNA. Oligos used to introduce the I-*Sce*I cleavage sequence at the *Mfe*I cutting site were 5′-AATTTAGGGATAACAGGGTAAT-3′and 5′-AATTATTACCCTGTTATCCCTA-3′.

For constructing *UAS-dRecQ4*, primers 5′-ATAAGAAT*GCGGCCGC*ACATGGACGATTCGGTGTTC-3′ and 5′-GG*GGT ACC*TCACGTACGCCTCTTGATAA3′ were used to PCR the genomic DNA that spans from the start to the stop codons of *dRecQ4* gene's coding region for putting into pUAST vector at *Not* I and *Kpn* I sites (start and stop codons are underlined).

*pTARG-dRecQ4[rescue]* construct contains 2.1 kb upstream of the ATG and 1.5 kb downstream after stop codon sequence in addition to the entire coding region. It was constructed by putting two PCR products into the *pTARG* vector at *Not* I and *Avr* II sites. The two pairs of primers used for PCR were as follows: 5′-AATGAATT*GCGGCCGC*GTCGGGAACTACAGTCCAACCT-3′/5′-CGAA*ACCGGT*TGGCTTAGGGAAGCTTCG-3′ and 5′-GCCA*ACCGGT*TTCGCAAGAGAAAGCAGC-3′/5′-TAGA*CCTAGG*ATGAAGGAGCACGGCCAAATGCCAG-3′. Restriction sites for cloning are highlighted in italics.

For primers that are used for generating domain dissection constructs, please see Table 3. Detailed cloning strategies are available upon request.

**Table 3:** Forward and reverse primers used to generate *pUAST-dRecQ4△* constructs that produce truncated proteins used in Fig. 8 #.

Construct	Primers
*pUAST-dRecQ4*	GGGctcgagATGGACTACAAAGACCATGA
	GGggtaccTTACTTGTCATCGTCATCCTTGT
*pUAST-dRecQ4△868-1579*	AAGGAAAAgcggccgcATGGACGATTCGGTGTTCAAGCT
	GGGctcgagCCCGAACATGTGGAGTGCCTCTA
*pUAST-dRecQ4△1234-1579*	AAGGAAAAgcggccgcATGGACGATTCGGTGTTCAAGCT
	GGGctcgagAGAATACACATGGCGACGCAGCT
*pUAST-dRecQ4△1-807*	AAGGAAAAgcggccgcATGACATACGTCGGCCACAAGATTCC
	GGGctcgagCGTACGCCTCTTGATAATAGCCA
*pUAST-dRecQ4△868-1207* *	ACGCgtcgacATGTTGCCTTCCCACTGTCACCTCTT
	GGGggtaccTTACTTGTCATCGTCATCCTTGT
*pUAST-dRecQ4△1-1207*	AAGGAAAAgcggccgcATGTTGCCTTCCCACTGTCACCTCTT
	GGGctcgagCGTACGCCTCTTGATAATAGCCA

# All proteins resulted from above constructs are Flag-tagged.

* For constructing *pUAST-dRecQ4△868–1207*, first PCR using *pUAST-dRecQ4△1–1207* as template, with primers listed as in the table, digested with *Sal* I and *Kpn* I, then ligated into *pUAST-dRecQ4△868–1579* vector which had been digested with *Xho* I and *Kpn* I.

### Fly stocks and genetics

Flies were cultured at 25°C for all experiments. For generation of germline clones (GLCs), we used the *FLP-DFS* system as described [57]. Briefly, the *dRecQ4^14^* mutation was recombined with the third chromosomal *FRT* insertion *2A*, balanced with *TM6B, Tb* balancer and crossed with *ovo^D^, FRT2A* males. Offspring of this cross were given a heat shock (37°C, 2 hrs) at late third instar larval stage and virgin females with correct genotypes (Table 1) were crossed with heterozygous mutant males.

Listed below are fly stocks used in this study:

1. *y w*
2. Canton S
3. *w*; *actin-Gal4*
4. *y w*; *ey-FLP; MKRS/TM2, y+*
5. *y w*;* hs-I SceI, hs-FLP, Sco/CyO*
6. *w1118*; *hs-I-CreI, Sb/TM6*
7. *FRT2A* (kindly provided by Dr. Xinhua Lin)
8. *y w, hs-FLP*; *FRT2A ovoD/TM3, Sb* (kindly provided by Dr. Xinhua Lin)
9. *y w*; *actin-Gal4/TM3, Ser*
10. *y w; pTARG-dRecQ4[rescue]*
11. *w1118; P{XP}d02769 P{neoFRT}80B* (Bloomington Drosophila Stock Center)
12. *y w, hs-FLP; If/CyO; Ubi-GFP FRT80B/TM6B, Tb (kindly provided by Dr. Zhaohui Wang)*
13. *Sp P[Rr3] 48C L/CyO* (Kindly provided by Dr. William R. Engels)
14. *Sco/CyO P[UIE] 53D* (Kindly provided by Dr. William R. Engels)

### Generation of *dRecQ4^14^* mutant

For generation of the *dRecQ4* mutant, we used the ends-in gene targeting method [47], [48]. Donor transgenic flies that bear the targeting construct on the second chromosome were crossed to flies that contain *hs-I SceI* and *hs-FLP* transgenes. Three heat shocks (38°C, 1 hr each) were applied on days 2, 3 and 4 after egg laying. Heat-shocked virgins were singly crossed to *y w; ey-FLP; MKRS/TM2, y+* males, and females were screened for targeted integration of targeting construct indicated by the *w^+^* marker. Reduction of two *dRecQ4* copies (one wild type and one mutant copy) by I-*Cre*I was performed by crossing the targeted alleles to *w1118; hs-I-CreI, Sb/TM6*. The offspring were given a single heat shock (36°C, 1 hr) at the third instar larval stage. *w^−^* males were crossed individually to *y w*; *actin-Gal4/TM3, Ser* to make stocks. The allele, we designated *dRecQ4^14^*, was further characterized by DNA sequencing for the intended mutations, the primers for PCR were 5′-TCCCAGCATGTGATAGTCTG-3′ and 5′-TCCTCAAGATTACCAG AGCTC-3′. The resulting data has been deposited in GenBank (accession number GQ128383).

### Generation of somatic clones

Loss-of-function somatic clones were induced using *FLP/FRT* mediated mitotic recombination [58]. To induce the clones, first instar larvae with correct genotypes were heat shocked for 1 hour at 38°C and then dissected at third instar larval stage. Mutant clones and twin spot areas were measured with confocal images using the histogram function of Adobe Photoshop.

### Immunohistochemistry

For BrdU labeling, wild type, mutant or rescued mutant larvae were dissected in PBS and then incubated in PBS containing 1 mg/ml BrdU (Sigma B-5002) for 30 min at 25°C. After three rinses with PBS, samples were fixed for 30 min in 4% paraformaldehyde followed by washing 3 times in PBST (PBS, 0.1% Triton X-100) and then treatment with 2M HCl for 30 min. After three washes in PBST, samples were incubated with mouse anti-BrdU (1∶100, ZYMED). TRITC-conjugated anti-mouse secondary antibody (Jackson ImmunoResearch Laboratories) was used with dilution of 1∶100 for 2 hrs at room temperature. Images were taken under a Leica DM6000 confocal microscope. More than 10 brains were examined per genotype. Rabbit anti-phospho histone H3 (Ser 10) antibody (1∶100) for detecting mitotic phase was purchased from Millipore. FITC-conjugated anti-rabbit secondary antibody (1∶100) was from Jackson ImmunoResearch Laboratories. Over 10 discs were analyzed for each genotype.

### Fluorescence activated cell sorting

80–100 wing discs of the same genotypes (the mutant or wild type) were dissected in PBS and digested with trypsin-EDTA (Sigma, T-4174). Cells were dissociated for 5 hours by gentle shaking. The dissociated cells were fixed in 75% ethanol, stained with propidium iodide and analyzed with a Becton Dickinson Vantage Fluorescence activated cell sorter. The events of either the wild type or the mutant were 10000. The experiment was repeated 3 times.

### DNA content measurement

Genomic DNA from 100 salivary glands of wild type or *dRecQ4^14^* mutant larvae (5 days old) was extracted, followed by A260/280 measurement. Two sample independent t-test was used to determine statistical significance.

### Chromosome spreads

Brains from wild type *Canton S* and *dRecQ4^14^* homozygous third instar larvae were dissected in 0.7% saline, treated with colchicine and hypotonic solution, fixed in acetic acid/methanol/H2O (11∶11∶2) and stained for 5 min in 0.2 µg/ml DAPI. The preparations were examined under Leica DM6000 fluorescent microscope.

### DNA damage sensitivity tests

Four mutagens were used in this experiment (hydroxyurea, methyl methane sulfonate, paraquat and γ-irradiation, see Table 2). All chemicals were purchased from Sigma. Chemical mutagens were added to fly food at final concentrations that have been used for DNA damage assay in *Drosophila* (see also Table 2). 20 females and 10 males of *y w*; *dRecQ4^14^*/*TM3, Sb,Kr-GFP* flies were put in the vial that contained different mutagens for 6 h before the parents were discarded. At the 6^th^ day, the heterozygous (GFP^+^) and homozygous (GFP^−^) mutants were scored. The survival ratio was measured as the number of GFP^−^ to half the number of GFP^+^. For γ-irradiation, eggs from *y w; dRecQ4^14^*/*TM3, Kr-GFP* flies were collected for 5 hrs and allowed to develop for 12 hrs before being exposed to 9 Gy γ-irradiation with a ^60^Co source. Only gamma irradiation was used to test the sensitivity of the rescued mutants to DNA damaging reagent. The same dosage was applied as above.

### DsRed repair assay

DsRed DSB repair model was carried out essentially according to the Preston method [46]. Since nearly all the *dRecQ4^14^* homozygous mutants can live to early pupal stage, the relative survival ratio from early instar larvae to early pupae stage was used to indicate the repair efficiency indirectly, assuming the lethality is caused by unrepaired DSBs. The cross was made as follows: *Sp P[Rr3] 48C L/CyO; dRecQ4^14^/TM3 Kr-GFP* crossed with *Sco/CyO P[UIE] 53D; dRecQ4^14^/TM3 Kr-GFP*. Offspring genotypes and phenotypes as judged by fluorescence colors are (homozygous *TM3 Kr-GFP* animals and homozygous *CyO* animals do not survive to pupae stage, therefore are not included for counting):

(A): *Sp P[Rr3]48C L/CyO P[UIE] 53D; dRecQ4^14^* [GFP (−) DsRed (+)]
(B): *Sp P[Rr3]48C L/Sco; dRecQ4^14^* and *CyO/Sco; dRecQ4^14^* [GFP (−) DsRed (−)]
(C): *Sp P[Rr3]48C L/CyO P[UIE] 53D; dRecQ4^14^/TM3, Kr-GFP* [GFP (+) DsRed (+)]
(D): *Sp P[Rr3]48C L/Sco; dRecQ4^14^/TM3, Kr-GFP* and *CyO/Sco; dRecQ4^14^/TM3, Kr-GFP* [GFP (+) DsRed (−)]

First instar larvae and early pupae representing each categories were counted for calculation of the relative survival ratio.
